# Cerebral small vessel disease: pearls and pitfalls from unexplored risk factors to treatment

**DOI:** 10.1055/s-0046-1829036

**Published:** 2026-10-06

**Authors:** Letícia Escorse Requião, Armando da Silva Rosa, Silvana Silva Macedo, Yasmin Curi de Almeida, Trajano Aguiar Pires Gonçalves, Iago Blanco Bacchiega, Rui Kleber do Vale Martins Filho, Maria Clara Zanon Zotin, Octávio Marques Pontes Neto

**Affiliations:** 1 Universidade de São Paulo, Faculdade de Medicina de Ribeirão Preto, Departamento de Neurociências e Ciências do Comportamento, Ribeirão Preto SP, Brazil.; 2 Universidade de São Paulo, Faculdade de Medicina de Ribeirão Preto, Departamento de Medicina Interna, Divisão de Radiologia, Ribeirão Preto SP, Brazil.

**Keywords:** Cerebral Small Vessel Diseases, Stroke, Cognitive Dysfunction, Clonal Hematopoiesis, Neuroimaging

## Abstract

Cerebral small vessel disease (CSVD) is a major cause of ischemic stroke and cognitive impairment, often accompanied by gait disturbances and neuropsychiatric symptoms that remain underrecognized. Age and hypertension are key risk factors, but emerging evidence points to the influence of genetic polymorphisms and clonal hematopoieses. The pathophysiology of CSVD is closely linked to neuroimaging features, as outlined in the Standards for Reporting Vascular changes on neuroimaging (STRIVE) criteria, which reflect the two most prevalent mechanisms: arteriosclerosis and cerebral amyloid angiopathy. As a clinico-radiological syndrome, CSVD encompasses imaging and histopathological findings. Despite its burden on function and quality of life, treatment remains limited to risk factor control. Ongoing studies aim to clarify the underlying mechanisms, validate biomarkers and identify novel therapeutic targets.

## INTRODUCTION

Cerebral small vessel disease (CSVD) encompasses all pathological processes affecting small arteries, arterioles, venules, and capillaries involved in cerebral circulation. 1 It is a common cause of stroke, accounting for ∼ 25% of ischemic strokes and representing the leading etiology of intraparenchymal hemorrhage. Moreover, CSVD is recognized as the second most prevalent cause of cognitive impairment. 2

The investigation of CSVD dates back to the 19th century, when studies by Virchow and Robin identified fluid-filled perivascular spaces—later termed *Virchow-Robin spaces* —which, when dilated, became a hallmark of this pathology. 3 In the 1960s, C. Miller Fisher further expanded the concept by describing cerebral lacunae and proposing their link to ischemic injury within the cerebral microvasculature. 4

Beyond stroke and cognitive decline, CSVD is associated with significant morbidity, including gait disturbances, increased fall risk, depression, urinary incontinence, parkinsonism, and apathy—many of which are frequently overlooked in clinical practice. 5 Apathy, in particular, is often underdiagnosed and may occur independently of depressive symptoms. 6 Other neuropsychiatric manifestations include delirium, directly associated with white matter lesions, as well as personality and behavioral changes. 7

Due to its heterogeneous presentation, the dependence on magnetic resonance imaging (MRI) for diagnosis, and the absence of universally standardized neuroimaging criteria, 8 the global prevalence of CSVD remains imprecisely defined. However, it is likely to be even higher in low- and middle-income countries, where the burden of vascular risk factors is substantial. 9

The current narrative review aims to revisit the definitions, risk factors, pathophysiological mechanisms, clinical manifestations, diagnostic approaches, and management strategies related to CSVD.

## RISK FACTORS

The prevalence of CSVD increases with age, with a clear correlation between aging and the severity of associated pathological markers. The risk approximately doubles after the age of 75. 10 White matter hyperintensities (WMHs) are present in around 5% of individuals at age 50, reaching nearly universal prevalence by age 90. Likewise, the prevalence of cerebral microbleeds rises from 6.5% at age 50 to 36% in individuals over 80 years. 11 12

Biological sex has not been established as a significant risk factor for CSVD. The influence of ethnicity and race remains incompletely understood. Some studies report a higher prevalence of confluent WMHs among individuals of Chinese ancestry, while the prevalence of lacunes and microbleeds appears similar across White and Black racial groups as well as among the individuals of Hispanic ethnicity. 13

Among modifiable factors, systemic arterial hypertension is the most important contributor to CSVD. 14 A cohort study with 668 individuals demonstrated its independent association with the progression of white matter lesions in patients aged 60 to 90 years. 15 Another study involving 414 White patients with CSVD found that systolic blood pressure > 140 mm Hg and diastolic pressure > 90 mm Hg were associated with a 3-fold increase in disease prevalence, 16 and higher BP variability is also associated with a higher burden of WMHs. 17 Other recognized risk factors include diabetes mellitus, hyperlipidemia, active smoking, obstructive sleep apnea, and chronic kidney disease. 18

Although rare, monogenic conditions can also lead to CSVD. The most prevalent inherited form is cerebral autosomal dominant arteriopathy with subcortical infarcts and leukoencephalopathy (CADASIL), with an estimated global prevalence of 2 to 5 cases per 100 thousand individuals. 19

Genetic association studies in both clinical and population-based cohorts have suggested that common, non-pathogenic variants in the *NOTCH3* and *COL4A1/A2* genes may also contribute to the more prevalent sporadic forms of CSVD. 20 In a cohort of 4,773 hypertensive individuals from the population-based Cohorts for Heart and Aging Research in Genomic Epidemiology (CHARGE) consortium, *NOTCH3* variants were associated with both the presence and progression of WMHs. 17 Furthermore, a case-control study involving over 1,500 individuals from the International Stroke Genetics Consortium demonstrated that common variants in the *COL4A1/A2* gene cluster were significantly associated with deep intracerebral hemorrhage. 19 These variants also showed associations with lacunar stroke and WMH burden among patients with symptomatic ischemic stroke. 19

Recently, acquired somatic mutations in hematopoietic progenitor cells—known as clonal hematopoieses—have emerged as a novel risk factor for CSVD. Clonal hematopoiesis of indeterminate potential (CHIP) refers to the age-related clonal expansion of hematopoietic stem cells harboring somatic mutations, most commonly in genes such as *DNMT3A* , *TET2* , *ASXL1* , and *JAK2* , in otherwise asymptomatic individuals. 21 Clonal hematopoiesis of indeterminate potential has been associated with increased susceptibility to small vessel disease, with a reported 1.55-fold higher prevalence of the susceptibility vessel sign on neuroimaging. These mutations are thought to promote pro-inflammatory and pro-atherosclerotic pathways, thereby contributing to the pathogenesis of CSVD. 22

Clonal hematopoiesis of indeterminate potential is related with increased systemic inflammatory markers, including high-sensitivity C-reactive protein and proinflammatory cytokines such as interleukin (IL)-1β and IL-6, and promotes hematopoiesis in both myeloid and lymphoid lineages. 23 Moreover, this inflammatory state is further driven by enhanced inflammasome activation and by leukocytes harboring CHIP-related mutations with increased self-renewal capacity. Collectively, these factors contribute to endothelial dysfunction, plaque progression, and an increased thrombotic risk. 24

## MECHANISMS

Cerebral small vessel disease encompasses distinct subtypes, which can be classified according to their etiopathogenic mechanisms into six categories: arteriosclerosis, cerebral amyloid angiopathy, genetic forms, inflammatory and immune-mediated CSVD, venous collagenosis, and other types. The latter includes, for instance, radiation-induced angiopathy and non-amyloid microvascular degeneration observed in Alzheimer's disease. 1

Among these, arteriosclerosis and cerebral amyloid angiopathy represent the two predominant mechanisms underlying CSVD. 2 Normal blood vessels may undergo pathological changes in response to various risk factors ( Figure 1A ).

### Arteriosclerosis

Arteriosclerosis is a systemic condition closely associated with aging, diabetes, and, most notably, hypertension. Hypertensive microangiopathy encompasses histopathological changes such as hyaline and hyperplastic arteriosclerosis, segmental arterial disorganization, and microaneurysm formation. 1 5

This vasculopathy is characterized by loss of smooth muscle cells in the tunica media, resulting in luminal narrowing and reduced cerebral blood flow. 1 These changes trigger a cascade of neurovascular unit dysfunction involving hypoxia, blood-brain barrier disruption, inflammation, and oligodendrocyte apoptosis. 23 The arteriosclerotic vessel demonstrates impaired autoregulation, predisposing to cerebral hypoperfusion and subsequent acute subcortical infarcts and chronic ischemia ( Figure 1B ).

**Figure 1:**
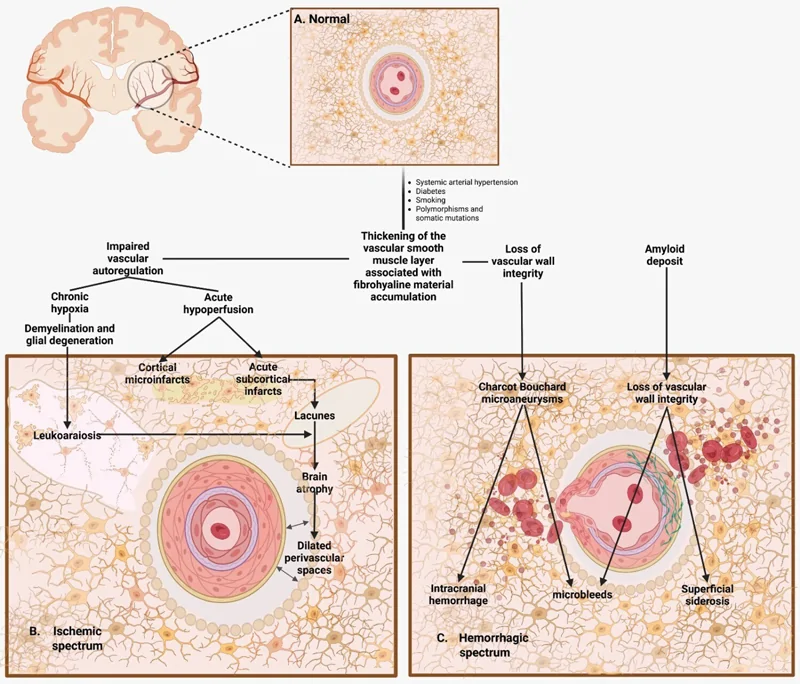
Schematic diagram for risk factors and pathogenesis of brain damage in cerebral small vessel disease. ( **A** ) Normal vessel; ( **B** ) ischemic changes in Cerebral small vessel disease (CSVD); and ( **C** ) hemorrhagic mechanisms in CSVD.

Acute ischemic injury in the territory of small vessels results in recent subcortical infarcts, which may evolve into round or ovoid, fluid-filled cavities measuring 3 to 15 mm, termed lacunes. 24 ^.^ Lacunar infarcts are primarily caused by lipohyalinosis, a destructive vasculopathy characterized by concentric hyaline thickening of small penetrating arteries, leading to their occlusion. This process, strongly linked to hypertension, involves fibrinoid necrosis and foam cell accumulation that contribute to luminal obstruction. 25 Lacunes measuring 3 to 7 mm on neuroimaging are typically attributed to lipohyalinosis, whereas those larger than 7 mm may suggest alternative etiologies such as atherosclerosis or embolism. 26

The enlargement of perivascular spaces, Virchow-Robin spaces, surrounding deep perforating arteries has been attributed to vascular ectasia and tortuosity, increased vessel wall permeability, reduced glymphatic clearance, and ex vacuo expansion due to cortical atrophy. 4 These spaces are generally smaller than 1 × 2 mm and commonly located in the centrum semiovale and basal ganglia. In contrast, lacunes are more frequently found in the basal ganglia, internal capsule, thalamus, and pons. 24

Chronic white matter hypoperfusion results in degeneration of myelinated fibers, driven by repeated oligodendrocyte death. This leads to rarefaction of the white matter, observed on imaging as leukoaraiosis ( Figure 1B ). The resultant demyelination and gliosis appear as hyperintensities on brain MRI, 25 while corresponding computed tomography findings reveal periventricular or subcortical hypodensities. 1

In addition, chronic hypertension may lead to the formation of Charcot-Bouchard aneurysms, which occur in small perforating arteries branching directly from large-caliber vessels such as the middle cerebral and basilar arteries. Due to their direct exposure to high-pressure flow, these vessels experience increased wall stress and muscular layer atrophy, favoring microaneurysm formation. 25 27

### Cerebral amyloid angiopathy

Another mechanism implicated in CSVD involves the pathological accumulation of proteins within the vascular wall, resulting in increased vessel fragility and a predominance of hemorrhagic manifestations. In this context, the deposition of amyloid protein plays a central role. 27 Cerebral amyloid angiopathy (CAA) is characterized by the progressive accumulation of β-amyloid in the walls of small and medium-sized cortical and leptomeningeal arteries and arterioles. 1 27 In more advanced stages, vascular dilation, wall disruption, and blood extravasation may occur, reflecting vessel degeneration. 27 28

In addition to large lobar hemorrhages, CAA is strongly associated with two hallmark imaging markers of CSVD: cerebral microbleeds and cortical superficial siderosis (cSS) ( Figure 1C ).

Cerebral microbleeds (CMBs) are small foci of hemosiderin deposition representing prior microhemorrhages. When associated with advanced amyloid angiopathy, CMBs often have a lobar distribution. 29 These lesions reflect previous blood leakage following the rupture of fragile vessels or microaneurysms. One study regarding neuropathologic findings in CAA showed that the majority of microbleeds corresponded to focal accumulations of siderophages. They were often accompanied by reactive astrocytes, which could indicate gliotic response to primary hemorrhagic injury. Nevertheless, in certain cases, reactive astrocytes were accompanied by pronounced tissue loss, an alternative hypothesis could be that this reflects primary ischemic tissue injury with hemorrhagic transformation. 30

Cortical superficial siderosis is defined by linear deposits of hemosiderin, particularly along the convexities of the cerebral hemispheres. 31 In individuals over 60 years, cSS is most commonly attributed to CAA and holds significant prognostic value. Its presence is associated with an increased risk of intracerebral hemorrhage, transient focal neurological episodes, and cognitive decline. 31 32

## CLINICAL SYNDROMES

### Vascular cognitive impairment

Vascular cognitive impairment and dementia (VCID) encompass a clinical spectrum ranging from mild cognitive deficits to advanced dementia. It may result from distinct pathophysiological mechanisms associated with CSVD, notably arteriosclerosis and CAA 32 33

Vascular disease is the second most common cause of dementia worldwide. 32 34 Following a stroke, the risk of cognitive impairment may increase up to 6-fold. 1 3 The prevalence of post-stroke cognitive impairment has been rising, partly due to improved survival rates and the growing burden of vascular risk factors. 1 Thus, cognitive assessment should be routinely performed in all poststroke patients, even in the absence of reported symptoms. 35

Mechanisms underlying vascular cognitive decline include strategic infarcts and multiple infarcts, often related to large vessel disease, which cause abrupt cognitive deterioration when critical cortical or subcortical areas (e.g., dominant frontotemporal cortex or thalamus) are affected. In contrast, small vessel disease leads to progressive microvascular damage, particularly in the subcortical white matter, resulting in cognitive decline even in the absence of overt stroke events. 32 33

Unlike the stepwise deterioration seen in large vessel disease, cognitive impairment in CSVD follows a more insidious and chronic course. It typically lacks focal neurological signs and a clear temporal relationship with vascular events. 1 This subtype, formerly referred to as subcortical dementia, predominantly affects executive functions and processing speed, but may also impair language, memory, attention, and visuospatial skills. 35 36 Early deficits in executive functioning and attention are characteristic, with memory impairment becoming more prominent in later stages. 2 35 36

Damage to diffuse subcortical neural networks, demonstrated by advanced neuroimaging techniques such as functional MRI and tractography, helps explain the cognitive profile observed in VCID. 32 34 35 Although executive dysfunction is the hallmark of CSVD-related impairment, memory deficits may be more prominent in patients with coexisting Alzheimer's disease pathology. 37 38 This overlap is common in clinical practice, particularly in older adults with multiple vascular risk factors, and is associated with a higher risk of developing clinical dementia. 38

### Lacunar stroke

Lacunar strokes are among the main clinical manifestations of CSVD, accounting for up to 25% of all ischemic strokes. 33 39 They typically result from the occlusion of small penetrating arteries. 9 Other mechanisms have been described, such as the obstruction of the ostium of perforating arteries by atherosclerotic plaque—known as branch atheromatous disease—or even by cardiac microemboli. Although the latter has been demonstrated in animal models, human studies suggest it plays a minor role in lacunar stroke pathogenesis. 33 39 40

The clinical presentation of acute subcortical infarcts depends on their location. However, due to the typical vascular territories of penetrating arteries, five classic lacunar syndromes have been described: pure motor syndrome, pure sensory syndrome, sensorimotor syndrome, ataxic hemiparesis, and dysarthria-clumsy hand syndrome. 40 Despite this, many lacunar infarcts are asymptomatic and incidentally detected on neuroimaging. 40 41

Another possible manifestation is capsular warning syndrome (CWS), characterized by multiple relapsing transient sensorimotor symptoms affecting the contralateral hemibody, in the absence of cortical signs. Capsular warning syndrome is associated with an elevated risk of completed stroke. 19

Lacunar infarcts may follow a progressive course in the acute phase. This progression is believed to result from a neurochemical imbalance, with increased glutamatergic activity leading to excitotoxicity, and decreased GABAergic activity contributing to neuronal vulnerability. 42 Although lacunar infarcts generally have a more favorable prognosis compared with other stroke subtypes, the progressive forms are associated with worse outcomes. 42

Less commonly, lacunar strokes may occur in younger individuals (under 60 years) and in the absence of conventional vascular risk factors. In such cases, hereditary forms of CSVD should be considered. A detailed family history and evaluation for systemic features are essential to guide diagnosis and management. 33

### Hemorrhagic stroke

Cerebral small vessel disease is the leading cause of intraparenchymal hemorrhage, also known as intracerebral hemorrhage (ICH), with deep hematomas—particularly those associated with systemic arterial hypertension—being the most common presentation. 43 44 These hemorrhages result from the rupture of Charcot-Bouchard microaneurysms in small perforating arteries, most commonly the lenticulostriate and pontine perforators. 43 The resulting vascular injury predisposes to bleeding in characteristic regions, including the putamen, caudate nucleus, thalamo-capsular region, pons, and dentate nucleus of the cerebellum. 43

Lobar hematomas, in contrast, are more frequently linked to CAA, which accounts for ∼ 50 to 57% of lobar ICH cases 45 However, ∼ 39% of lobar hematomas occur in association with arteriosclerosis, typically due to hypertension, even in the absence of CAA. 44 Hemorrhages located in mixed or overlapping regions are more commonly attributed to hypertensive small vessel disease. 44 Nonetheless, other CSVD-related etiologies involving vascular fragility—particularly CAA—should always be considered. 34

Certain monogenic forms of CSVD, unrelated to amyloid angiopathy, may also increase the risk of hemorrhage. Missense or null variants in the *COL4A1* and *COL4A2* genes confer the highest hemorrhagic risk. 46 Moreover, patients with CADASIL and other genetically determined ischemic CSVD may be susceptible to hemorrhage, particularly in the context of poorly controlled hypertension or anticoagulant use. 47

Recent clinical trials have provided new insights into the acute management of ICH. The Intensive Care Bundle with Blood Pressure Reduction in Acute Cerebral Hemorrhage Trial (INTERACT-3) study demonstrated that a bundled strategy, including early blood pressure control, reversal of coagulopathy, temperature regulation, and glycemic management, can significantly improve 6-month functional outcomes, as measured by a favorable shift in the modified Rankin scale. 48 Similarly, the Early Minimally-Invasive Removal of Intracerebral Hemorrhage (ENRICH) trial showed that early (within 24 hours) minimally invasive surgical evacuation of hematomas is associated with better functional outcomes at 6 months. 49 These findings represent a paradigm shift in the treatment of ICH, a condition that previously lacked effective evidence-based interventions.

### Vascular parkinsonism

There is a well-documented association between cerebrovascular disease and parkinsonism, initially described by Critchley in 1929 as *arteriosclerotic parkinsonism* , now more commonly referred to as vascular parkinsonism (VP). 50 This secondary form of parkinsonism accounts for ∼ 3 to 6% of all parkinsonian syndromes and has a prevalence of 2.3% in the elderly population. 51

Clinically, VP is characterized by gait disturbance, including freezing of gait, postural instability, frequent falls, and bradykinesia predominantly affecting the lower limbs. 52 In the context of cerebral small vessel disease, these symptoms usually develop gradually and chronically, resulting from diffuse white matter dysfunction. However, VP may also emerge acutely following strategic lacunar infarcts in basal ganglia regions. 52 53

Certain clinical features favor a vascular etiology over idiopathic Parkinson's disease, including older age at onset, shorter disease duration, lower limb predominance, bilateral and symmetrical motor signs, and poor response to levodopa. 52 53 54 Corticospinal tract involvement may also occur, manifesting as pyramidal signs, thus forming part of the non-inherited parkinsonism-pyramidal syndrome, which can further complicate differential diagnosis. 54

Neuroimaging in VP typically reveals WMHs, periventricular changes, and microinfarcts in deep structures such as the basal ganglia, thalamus, substantia nigra, or claustrum. 43 46 55 Dopamine transporter imaging (e.g., DaTscan) can aid in distinguishing VP from idiopathic PD, as VP often shows preserved presynaptic dopaminergic function.

Despite these findings, the diagnosis of VP remains controversial due to the uncertain causal relationship between vascular changes and parkinsonian symptoms. This ambiguity is partly due to the low specificity of neuroimaging markers—WMHs and lacunes can be seen in other conditions such as normal pressure hydrocephalus or even in asymptomatic elderly individuals. 56

Consequently, some authors argue that VP lacks a specific structural imaging pattern. Clinicopathologic correlation is often poor, and a subset of patients initially diagnosed with VP may ultimately be reclassified as having neurodegenerative parkinsonian syndromes, such as idiopathic Parkinson's disease or progressive supranuclear palsy, which may also present with nonspecific imaging findings. 55

### Other neuropsychiatric manifestations

Cerebral small vessel disease is frequently associated with a range of neuropsychiatric symptoms that are often overlooked or underdiagnosed, particularly in patients with prominent motor deficits or cognitive impairment. These manifestations include depressive symptoms, apathy, emotional lability, fatigue, personality changes, and a heightened susceptibility to delirium. 34 36 43 White matter hyperintensities—one of the hallmark imaging features of CSVD—are independently associated with the presence and severity of these clinical manifestations. 53

Apathy is among the most prevalent neuropsychiatric symptoms in CSVD, affecting ∼ 48% of patients. It may occur independently of depression and is increasingly recognized as a distinct clinical entity. 33 Fatigue is also common, present in ∼ 46% of stroke survivors and particularly frequent in CSVD, especially when accompanied by comorbid depression. 51 Delirium may occur following various cerebrovascular insults, but several studies have demonstrated a higher likelihood in patients with underlying CSVD. 57

The pathophysiology of these symptoms is closely linked to the disruption of subcortical white matter tracts. Progressive WMHs are associated with worsening of neuropsychiatric symptoms, suggesting a shared underlying mechanism. 58 Nevertheless, certain symptoms may correspond to specific patterns of structural damage—for example, apathy has been associated with lesions affecting prefrontal, parietal, thalamocortical, and striatocortical circuits. 34 57 58 Depression, though pathophysiologically more complex and difficult to manage, may also be related to white matter disconnection. 59

The role of inflammation in neuropsychiatric symptoms remains uncertain; however, some evidence suggests that systemic inflammation may contribute to fatigue. 60

There is currently limited evidence supporting targeted treatment for these symptoms in the context of CSVD. Management typically follows standard approaches used in other clinical settings. 61 A randomized controlled trial involving 41 patients tested 400 mg of modafinil versus placebo and showed improvements in fatigue severity and quality of life. 51 However, a meta-analysis of 10 randomized trials did not find consistent evidence supporting any specific pharmacological treatment for post-stroke fatigue, highlighting this symptom as a persistent therapeutic challenge. 61

## NEUROIMAGING

In CSVD, neuroimaging markers that characterize small vessel injury and its consequences in brain tissue are fundamental to its definition. These markers can be identified through various cranial MRI sequences, making CSVD a clinical-radiological syndrome. To standardize and organize the interpretation of these findings, the STRIVE study was published in 2013 and later revised in 2023.

Currently, the imaging markers used in the evaluation of patients with CSVD include recent small subcortical infarcts, lacunes of presumed vascular origin, WMHs of presumed vascular origin, enlarged perivascular spaces, cerebral microbleeds, cortical superficial siderosis, and cerebral atrophy. 62

Recent small subcortical infarcts correspond to ischemic lesions in the territory of a single penetrating arteriole, with clinical or imaging features indicating occurrence within the past few weeks (approximately three weeks). The STRIVE study defines these lesions as measuring up to 20 mm in maximum axial diameter to operationalize their correspondence to the territory of a penetrating arteriole. 62 These infarcts should be associated with compatible clinical symptoms, typically focal neurological deficits. Asymptomatic lesions or those associated with atypical symptoms—referred to as “hidden lesions”—are not currently considered a core feature of CSVD but are regarded as emerging findings, as their mechanisms are not yet fully understood. On MRI, the lesion typically appears hyperintense on T2-weighted, fluid-attenuated inversion recovery (FLAIR), and diffusion-weighted imaging (DWI) sequences, and hypointense on the apparent diffusion coefficient (ADC) map. Over time, lesion evolution varies; however, progression to cavitation and formation of lacunes is the most common outcome. Other possible residual patterns include partial cavitation, lacunes smaller than 3 mm, persistent hyperintensity on T2 and FLAIR, transient or permanent disappearance of the lesion, residual hemosiderin deposition, or a hemosiderin rim (seen as hypointensity on T2 sequences). 63

Lacunes of presumed vascular origin are defined as round or ovoid subcortical cavities filled with cerebrospinal fluid (CSF), typically exhibiting CSF-isointense signal across all MRI sequences. They measure up to 15 mm in diameter and are usually identified on visual assessment of T1-, T2-, and FLAIR-weighted images. 62 Lacunes are more frequently observed in the centrum semiovale and basal ganglia. A study published in 2016, which analyzed 88 lacunes in 57 genetically confirmed CSVD patients using three-dimensional MRI, found that ∼ 10% of lesions exceeded 15 mm in diameter on volumetric imaging; however, only ∼ 1% did so when assessed on conventional axial sequences, supporting the clinical validity of the 15 mm cut-off in standard imaging protocols. 64

It is essential to distinguish lacunes from perivascular spaces. Lacunes typically appear on FLAIR as hypointense cavities with or without a hyperintense rim. However, perivascular spaces traversing hyperintense white matter regions may mimic this rim. Lacunes generally measure between 3 and 15 mm, whereas cavities smaller than 3 mm are more likely to correspond to perivascular spaces, although no definitive cut-off has been established for differentiation. 65 The presence of lacunes is associated with increased risk of stroke, dementia, and gait impairment, 4 as well as with poorer long-term outcomes in patients with a history of stroke. 8

Enlarged perivascular spaces are fluid-filled cavities that exhibit CSF-like signal across all MRI sequences and follow the anatomical trajectory of penetrating vessels through white or deep gray matter. Morphologically, they are usually round or ovoid but may appear linear depending on the imaging plane orientation. When evaluated on slices perpendicular to the vessel path, their diameter typically does not exceed 2 to 3 mm. 65 Although perivascular spaces can be visualized on T1-weighted images, high-resolution T2-weighted sequences are more sensitive for their detection. 65 66 Their distribution pattern may reflect different pathophysiological processes. Perivascular spaces located in the centrum semiovale are more commonly associated with cerebral amyloid angiopathy and are part of the Boston criteria, whereas those located in the basal ganglia are more frequently linked to arteriolosclerosis. 67 A prospective cohort published in 2017, including 229 patients with intracerebral hemorrhage due to amyloid angiopathy, identified a high burden of perivascular spaces in the centrum semiovale as an independent predictor of hemorrhagic recurrence. 68

White matter hyperintensities of presumed vascular origin are characterized by signal alterations within the cerebral white matter, varying in size and typically distributed symmetrically between the hemispheres. These lesions appear as hyperintense areas on T2-weighted and FLAIR sequences. 65 Although commonly observed in older adults, their severity correlates strongly with cerebrovascular disease and vascular risk factors. Automated methods for the analysis and quantification of WMHs are currently under development but remain insufficiently validated. Therefore, visual rating scales are still essential, such as the one proposed by Fazekas et al. 68 ( Table 1 ).

**Table 1:** Fazekas et al. ^68^ Scale for White-Matter Lesions

1. Periventricular hyperintensity: 0: absent; 1: “caps” or pencil-thin lining; 2: smooth “halo;” and 3: irregular periventricular hyperintensity extending into the deep white matter 2. Deep white-matter hyperintensity: 0: absent; 1: punctate foci; 2: beginning confluence of foci; and 3: large confluent areas

Cerebral microbleeds are defined as small, round or ovoid areas measuring up to 10 mm in diameter—most commonly between 3 and 5 mm—characterized by marked hypointensity on iron-sensitive MRI sequences, such as gradient-echo T2 and susceptibility-weighted imaging (SWI). 65 Microbleeds located in the cortical gray matter and just-cortical white matter are classically related to amyloid angiopathy, while those located in deeper structures are more related to arteriolosclerosis. 69 In patients over 65 years old, strictly lobar microbleeds have good predictive value for amyloid angiopathy, as noted in the Boston criteria. 70 Post-mortem studies have shown a strong correspondence between neuroimaging findings and microbleeds in histopathological analysis. 71 The most commonly found lesions were acute microhemorrhage, hemosiderin residues from old hemorrhages, and small lacunae surrounded by hemosiderin. The presence of microbleeds has also been shown to be an independent predictor of cognitive decline. 72

Superficial cortical siderosis is identified as linear areas of marked hypointensity on iron-sensitive MRI sequences, such as gradient-echo T2 and SWI. These signal alterations are located in the superficial layers of the cerebral cortex or extend over its surface, and may be confined to specific sulci and gyri or present in a more diffuse distribution. 62 They correspond to radiological evidence of chronic hemoglobin derivatives, resulting from convexity subarachnoid hemorrhage (especially in older patients), hemorrhagic transformation of ischemias, bleeding from vascular malformations, or trauma. 62 65 Like microbleeds, cSS is also considered a diagnostic marker for amyloid angiopathy and a key feature of the Boston criteria. 71 The presence of superficial cortical siderosis is also pointed out as a strong predictor of clinical events in patients with amyloid angiopathy, such as ICH and cognitive and/or functional decline. 63

Cerebral atrophy refers to a generalized reduction in brain volume not attributable to specific macroscopic focal lesions, such as those resulting from trauma or infarction. It represents a nonspecific marker of cerebral small vessel disease and may coexist with atrophy related to other neurodegenerative processes. 63 Both cortical and subcortical atrophies can result from small vessel disease; however, its spatial distribution pattern still needs to be clarified. 73 There is also considerable variability in measuring cerebral atrophy, and there is no consensus on the interference of focal lesions. In this context, STRIVE-2 suggests that, ideally, atrophy should be referred to through serial assessments, and that quantification should only be performed after the stability of any acute lesions. 63 Magnetic resonance imaging findings are summarized in Figure 2 .

**Figure 2:**
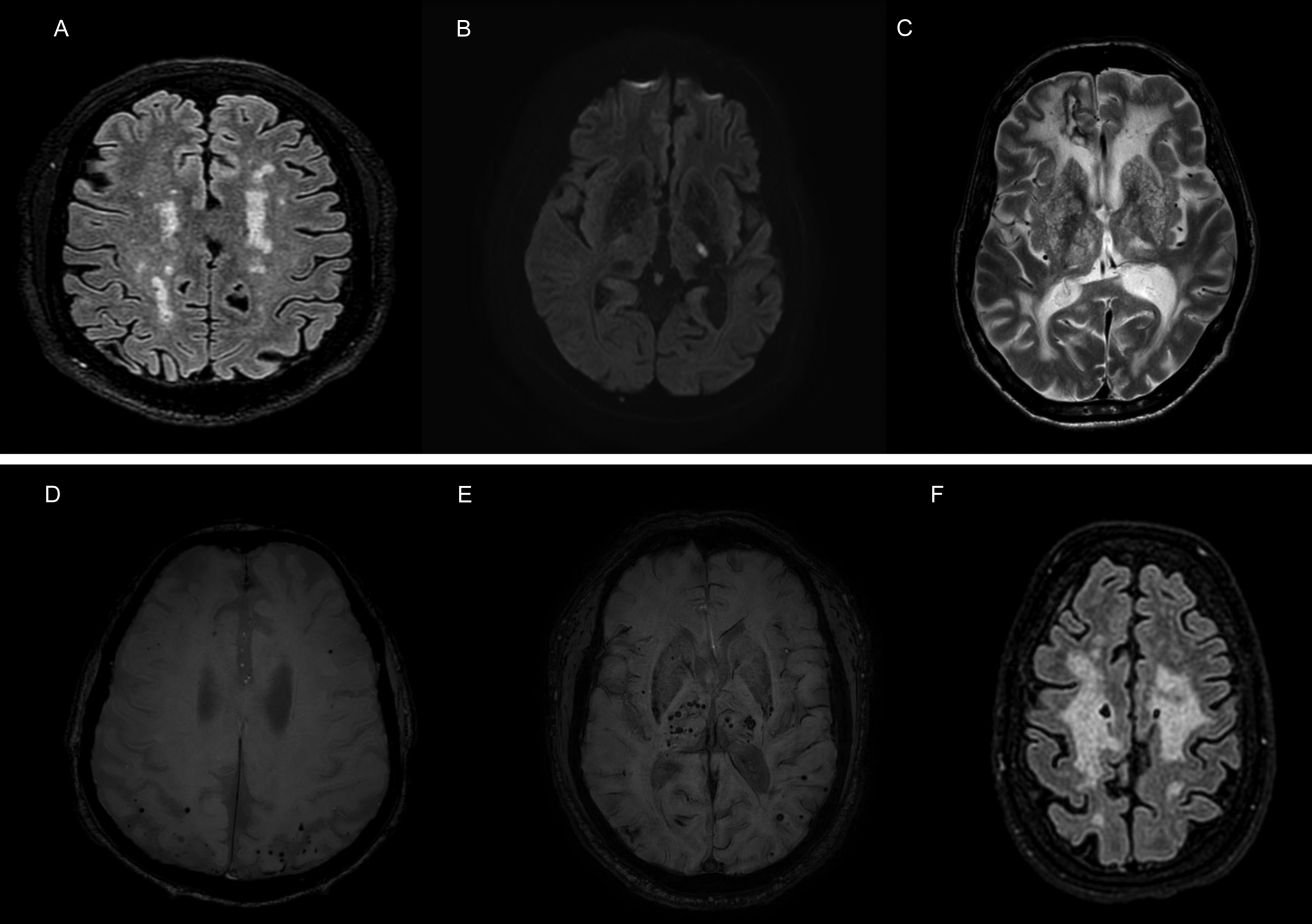
Abbreviations: FLAIR, fluid-attenuated inversion recovery; DWI, Diffusion-weighted imaging; MRI, magnetic resonance imaging; SWI, susceptibility weighted imaging. Neuroimaging features of cerebral small vessel disease on MRI. ( **A** ) Axial T2 FLAIR showing typical white-matter hyperintensities; ( **B** ) recent small subcortical infarct on axial DWI; ( **C** ) enlarged perivascular spaces intracerebral; ( **D** ) cortical superficial siderosis; ( **E** ) cerebral microbleeds in the cortex of a patient with possible cerebral amyloid angiopathy as shown on a SWI MRI sequence; and ( **F** ) lacunar infarcts in the high convexity on a T2 FLAIR.

## DIAGNOSTIC CHALLENGES AND PITFALLS IN THE DIFFERENTIAL DIAGNOSIS OF CSVD

The diagnostic approach to patients with CSVD may arise in multiple clinical settings, ranging from acute presentations, such as intraparenchymal hematomas or lacunar strokes, to outpatient scenarios often characterized by nonspecific complaints. In this context, the differential diagnosis becomes particularly challenging, especially in younger patients or in the presence of extensive white matter disease, situations in which the likelihood of alternative diagnoses increases.

A particularly challenging scenario is the evaluation of young patients (< 60 years) presenting with a high burden of white matter disease. In these situations, a systematic search for clinical and radiological findings supporting the hypothesis of CSVD is essential, as the probability of diagnostic misclassification is higher. In an observational study involving patients with symptoms suggestive of multiple sclerosis, 6% were found to have vascular white matter disease as the initial diagnostic hypothesis. 71

In this setting, careful attention to characteristic neuroimaging findings becomes crucial. In multiple sclerosis, for example, the radial perivenular distribution of demyelinating lesions, frequently extending to the calloso-septal interface, is a notable feature and is extremely uncommon in CSVD. On SWI sequences, the identification of the central vein sign and paramagnetic rim lesions is not uncommon, findings that have greater specificity for multiple sclerosis compared with CSVD. In contrast, SWI frequently reveals microbleeds and cortical siderosis in patients with CSVD, findings that are not part of the typical radiological spectrum of multiple sclerosis. Gadolinium enhancement may occur in both conditions: in CSVD, it usually occurs in the context of subacute ischemic events, whereas in demyelinating diseases, it reflects inflammatory disease activity. However, peripheral open-ring enhancement, which is characteristic of multiple sclerosis, is not observed in CSVD. Furthermore, the presence of demyelinating-appearing lesions along the optic nerve pathway and within the spinal cord white matter also directs the diagnostic evaluation toward multiple sclerosis.

Within the brainstem, differences in lesion distribution may assist in the differential diagnosis. Lesions associated with CSVD are typically located in the pons, predominantly in its central portion, whereas in multiple sclerosis, they tend to have a more peripheral location, often touching the surface of the brainstem and following the course of cranial nerves, such as the trigeminal nerve.

Another important differential diagnosis in these scenarios includes genetic leukodystrophies, particularly those with adult onset that may present with asymmetric progression, such as *CSF1R* -related disorders, adult-onset Alexander disease, and metachromatic leukodystrophy. The presence of positive family history, consanguineous marriage, involvement of the peripheral nervous system—especially in the form of generalized areflexia—and progressive ataxic syndrome in the absence of clear cerebellar lesions should raise suspicion for alternative diagnoses related to adult leukodystrophies. 72

When imaging findings typical of CSVD are observed in patients younger than 60 years, particularly when they are extensive and associated with clinical manifestations such as cognitive impairment, lacunar or hemorrhagic events, even in the presence of cardiovascular risk factors, investigation of genes associated with hereditary forms of small vessel disease is recommended. 73 In this context, broad genetic sequencing approaches, such as whole-exome sequencing, are preferable to targeted testing of individual genes.

Finally, the proper identification of genetic or inflammatory causes of white matter disease has important clinical implications, as it allows both the early initiation of disease-modifying therapies and the implementation of appropriate genetic counseling.

## MANAGEMENT

Given the growing relevance of CSVD in the global health landscape, recent research efforts have focused on elucidating its underlying pathophysiology. These investigations have explored molecular mechanisms related to endothelial dysfunction, nitric oxide synthesis, blood-brain barrier integrity, extracellular matrix homeostasis, and reparative processes, with the aim of expanding therapeutic and preventive strategies for the clinical spectrum of CSVD. 2

Despite these advances, there is still limited evidence supporting effective preventive or therapeutic interventions specifically tailored to CSVD. 2 Prophylactic strategies for lacunar stroke, for instance, are mostly extrapolated from broader studies on ischemic stroke. Systemic arterial hypertension remains the principal modifiable risk factor associated with the onset and progression of CSVD. The most robust evidence for primary prevention derives from the SPRINT-MIND trial, which demonstrated that intensive blood pressure control (targeting systolic pressure < 120 mm Hg) reduced white matter hyperintensity progression and improved outcomes in patients with early cognitive impairment. 74 Conversely, there is a paucity of studies specifically assessing the effects of statin use, smoking cessation, glycemic control, or lifestyle interventions in the context of lacunar stroke. Nonetheless, the overall control of cardiovascular risk factors has been consistently associated with better functional outcomes and lower incidence of vascular events. 75

Regarding secondary prevention of lacunar infarcts, the Secondary Prevention of Small Subcortical Strokes (SPS3) trial remains the only large-scale randomized controlled study targeting patients with MRI-confirmed lacunar stroke. It demonstrated that strict blood pressure control, with a systolic target below 130 mm Hg, significantly reduced recurrence rates, particularly for hemorrhagic strokes. 76 Additionally, the study found that antiplatelet monotherapy was not inferior to dual antiplatelet therapy (DAPT). A 2015 systematic review analyzing two clinical trials concluded that antiplatelet monotherapy was superior to no treatment in reducing both ischemic and combined ischemic/hemorrhagic recurrences in patients with recent lacunar stroke. 77 Further studies incorporating MRI-based inclusion criteria are ongoing to better define the role of antithrombotic therapies in this population.

Several pharmacologic agents are currently under investigation. DL-3-n-butylphthalide, for example, has shown potential in improving cognitive performance as measured by the Montreal Cognitive Assessment (MoCA) and Mini-Mental State Examination (MMSE) scales. Its proposed mechanisms include attenuation of oxidative stress, inhibition of apoptosis, and enhancement of neuroplasticity. 78 Minocycline, known for its antiinflammatory effects in the central nervous system, has demonstrated the ability to reduce microglial activation and stabilize the blood-brain barrier. These effects may be monitored via positron emission tomography-computed tomography (PET-CT) and MRI, although more robust evidence is still required. 78

The multicenter, prospective Determinants of Incident Stroke Cognitive Outcomes and Vascular Effects on Recovery (DISCOVERY) study followed stroke survivors, many of them with lacunar infarcts, over a minimum of 2 years, with serial cognitive and functional assessments to identify risk factors associated with long-term dementia. 79 Similarly, the phase 2 Lacunar Intervention Trial-2 (LACI-2) evaluated the use of isosorbide mononitrate and cilostazol on vascular, functional, and cognitive outcomes in patients with lacunar stroke. While it did not demonstrate definitive therapeutic benefit, its results support further exploration in phase 3 trials. 80

In clinical practice, the management of symptomatic lacunar stroke includes secondary prevention with antiplatelet therapy and high-intensity statins. For patients with minor stroke, dual antiplatelet therapy (DAPT) is recommended for 21 days, based on trials showing reduced recurrence within the first 90 days. However, individuals with CSVD were underrepresented in these studies. 81

In patients with CSVD who also present high-risk cardioembolic sources, left atrial appendage occlusion may be considered as an alternative to long-term oral anticoagulation, especially in the presence of neuroimaging markers such as cortical superficial siderosis and cerebral microbleeds, which are associated with increased risk of intracranial hemorrhage. When anticoagulation is indicated, apixaban is generally preferred over other direct oral anticoagulants due to its more favorable safety profile regarding intracranial bleeding. 82 83 84

In conclusion, cerebral small vessel disease is an established cause of age-related disability. Until recently, limited attention had been given to emerging risk factors such as clonal hematopoiesis and genetic polymorphisms, although a better understanding of these mechanisms may contribute significantly to future epidemiological and therapeutic advances. The development of standardized radiological criteria has improved the ability to estimate the true prevalence of CSVD, which may be underestimated, particularly in low- and middle-income countries. Despite its clinical relevance, evidence on optimal management strategies remains limited. Ongoing research aims to clarify the underlying pathophysiology, validate biomarkers and to identify more effective preventive and therapeutic approaches for this heterogeneous and highly prevalent condition.
